# Comparative Study between Plastic and Metallic Stents for Biliary Decompression in Patients with Distal Biliary Obstruction

**DOI:** 10.1155/2017/7621821

**Published:** 2017-09-06

**Authors:** João Guilherme G. Cabral, Eloy Taglieri, Adriane Pelosof, Daniel Rosendo, José Celso Ardengh

**Affiliations:** ^1^Endoscopy Service of Hospital A. C. Camargo, São Paulo, SP, Brazil; ^2^Endoscopy Service of Hospital 9 de Julho, São Paulo, SP, Brazil; ^3^Department of Diagnosis by Image, The Paulista School of Medicine, UNIFESP, Diadema, SP, Brazil

## Abstract

This paper presents a retrospective comparison of plastic versus metallic stents in the drainage of malignant distal biliary obstructions. We compared single plastic stents (SPS), multiple plastic stents (MPS), and metallic stents (SEMS) regarding clinical decrease of TB < 2.0 mg/dL, long-term patency, and adverse event. 58 patients (38 women) with MDBO were included. Diagnoses were 44 pancreatic adenocarcinoma (74.6%), 9 metastasis (15.5%), 3 pancreatic neuroendocrine tumors (5.1%), and 2 adenocarcinoma in the major papilla (3.4%). The number of patients included in the SPS, MPS, and SEMS was 17, 6, and 35, respectively. Comparing the survival curves with respect to obstruction, we observed a lower mean permeability of the SPS compared to that of the MPS with *p* < 0.003 and of the SEMS group (*p* < 0.01). There was no statistical difference between the use of MPS, despite the small number of patients compared to the use of SEMS (*p* < 0.13) to reach the satisfactory levels of bilirubin.

## 1. Introduction

Patients with malignant distal biliary obstruction (MDBO) usually present with obstructive jaundice. These tumors behave aggressively and develop distant metastasis in high frequency, even in its earliest stages, thus decreasing the chance of curative resection. Biliary drainage is indicated to allow better life quality, usually as a palliative measure. Biliary drainage can be achieved by surgery, percutaneous interventions, and endoscopic retrograde cholangiopancreatography (ERCP). It is consensus that the most effective technique to reach biliary decompression is through ERCP because of a better safety profile, being less likely that complications occur. It also has a faster recovery after drainage when compared to that of surgery. It is possible to use plastic or metallic stents to overcome the malignant stenosis. Variations of the technique include the use of a single plastic stent (SPS), multiple plastic stents (MPS), or one self-expandable metallic stent (SEMS). SPS and MPS have mean patency time of 3 and 9 months, respectively [1]. MPS is most indicated in cases of postsurgical benign biliary strictures, mainly after orthotopic liver transplant.

Palliative or neoadjuvant chemotherapy has better results when there is no biliary obstruction because most of the chemotherapeutic agents are hepatotoxic. The association of oxaliplatin, 5-fluoracil, leucovorin, and irinotecan (FOLFIRINOX) is the palliative regimen that carries the best long-term survival in these patients but has the major side effect of causing steatohepatitis and periportal fibrosis with hepatic failure if the patient keeps jaundiced. Thus to decrease complication rate and improve efficacy, total bilirubin (TB) levels should be under 2.0 mg/dL prior to chemotherapy. When this TB level is not reached, alternative regimens using oxaliplatin without irinotecan or gemcitabine are available, but results are not as good as for FOLFIRINOX. The authors demonstrate and evaluate which endoscopic technique is the best to achieve satisfactory TB to start chemotherapy in patients with MDBO, to provide improvements in life quality. There is only one reference in the literature in regard to this theme, and this is the reason for this study to be published [2]. The main objective of this study was to evaluate and compare which technique of biliary stent interposition/implant (SPS, MPS, or SEMS) would be the best option to decrease TB levels under 2.0 mg/dL thus allowing the use of chemotherapy without restrictions.

## 2. Methods

Patients diagnosed with MDBO, submitted to biliary drainage through ERCP in the AC Camargo Cancer Center, São Paulo, Brazil, from January to December of 2015, were followed and their medical record revised from the staging diagnosis indicating endoscopic drainage until a new biliary obstruction occurred or death. We evaluated the clinical decrease of TB < 2.0 mg/dL, time to reach this level, long-term patency (time from intervention until obstruction or death) determined by increment in TB, need for new drainage, or occurrence of adverse event related to insufficient biliary drainage. We considered stent disfunction when any of the fallowing circumstances happened: jaundice recurrence, cholangitis, or asymptomatic raising in the hepatic enzymes, bilirubins, and ductal enzymes in conjunction.

### 2.1. Inclusion and Exclusion Criteria

We included patients diagnosed with MDBO by computed tomography (CT) and/or magnetic resonance (MR) not amenable to surgery and with life expectancy ≥ 4 months. We excluded those with previous biliary drainage, initial TB ≤ 2.0 mg/dL, and synchronic hilar strictures.

### 2.2. Endoscopic Biliary Drainage

All procedures were performed by experienced endoscopists (ET and AMS). We used straight plastic stents of 8.5 and 10 Fr (Cotton-Leung® Cook Medical, Limerick, Ireland) and metallic stents of 10 mm of diameter (WallFlex Biliary RX® Boston Scientific, Marlborough, Massachusetts, USA). The choice among SPS, MPS, and SEMS was left at the endoscopist's discretion. We compared three groups: group 1 (SPS), group 2 (MPS), and group 3 (SEMS).

### 2.3. Clinical Success, Adverse Events, and Follow-Up

Clinical success was considered when the TB level decreased below 2.0 mg/dL within the same hospital stay and unsuccessful when they do not. After initial treatment failure, the patients were submitted to one of the following: new endoscopic drainage, percutaneous drainage, or surgical drainage. Early adverse events (within 7 days) were registered and studied as well as the late ones (after 7 days of intervention). Their treatment was chosen and the results were also evaluated.

Our routine for patients with MDBO unfit for surgical resection is endoscopic drainage and palliative chemotherapy within the same hospital stay. On the first week, BT was controlled daily. After this period, BT levels were measured weekly during the chemotherapy cycles or according to the assisting clinical oncologist discretion, based on patients' clinical findings.

### 2.4. Statistical Analysis

The differences on demographic characteristics among groups SPS, MPS, and SEMS were made using independence analysis. Age comparison was made by the Kruskal-Wallis method and the qualitative variables by the Fisher's exact test. Patency time of stents used in each group was evaluated using the Kaplan-Meier survival curve, and a comparison among each other was made with the Mantel-Cox log-rank method. Stent patency time was considered the period from intervention until stent dysfunction or death.

## 3. Results

After applying exclusion criteria, 58 patients with MDBO were included in the final analysis. A total of 82 ERCP, median 1.4 procedure per patient (1–4), were made. There were 20 men and 38 women, with the median age of 64.5 y old (17–96). Final diagnoses were the following: 44 pancreatic adenocarcinoma (74.6%), 9 metastasis (15.5%), 3 pancreatic neuroendocrine tumors (5.1%), and 2 adenocarcinoma of the major duodenal papilla (3.4%).

The 3 groups were separated and evaluated according to drainage technique previously described in these study methods. There was no significant statistical difference among groups related to sex, age, and indications. Demographic data and indications separated by groups are shown on Table 1.

### 3.1. Group Analysis

#### 3.1.1. Single Plastic Stent (SPS)

Seventeen patients received this type of treatment. There were inserted with 10 Fr (14 (82%)) and 8.5 Fr (3 (18%)) stents. The mean patency was 70.5 (2–213) days. Clinical failure (total bilirubin remaining >2 mg/dL) was observed in 11 (64%) patients. New ERCP was indicated in 9 (81.8%) and 2 (18.2%) patients were referred for percutaneous biliary drainage. The overall rate of adverse events was 5/17 (29.4%). Early adverse events (up to 7 days) were identified in 3/17 (17.6%) patients (cholangitis (1), bleeding (1), and acute pancreatitis (1)). Cholangitis was the most frequent late adverse event, occurring in 2/17 (11.7%). All identified adverse events was treated conservatively. The 3 patients with cholangitis were treated by endoscopy, and patients with acute pancreatitis and bleeding were identified as mild and/or self-limited, requiring no endoscopic or surgical intervention.

#### 3.1.2. Multiple Plastic Stents (MPS)

This group consisted of 6 patients. The average number of stents used per patient was 2.5 (2-3), varying between 7 Fr (8), 8.5 Fr (4), and 10 Fr (3). The mean permeability time was 297 (3–534) days. Clinical failure occurred in 1 (16.6%) patient. The overall rate of adverse events was 1/6 (16.6%) patients, being the same patient with clinical failure. This patient had cholangitis 4 days after the procedure and percutaneous drainage was indicated.

#### 3.1.3. Self-Expandable Metallic Stent (SEMS)

Thirty-five patients underwent SEMS insertion. SEMS used were partially covered (20), uncovered (10), and totally covered (5). The mean SEMS patency was 190 (3–586) days. Clinical failure occurred in 10 (29%) patients. The overall rate of adverse events was 8.5% (3/35). Mild bleeding occurred in 2 (5.6%) and cases and acute pancreatitis in 1 (2%). All occurred within 7 days of the procedure. All were treated conservatively.

### 3.2. Comparison between Clinical Success and Survival

The mean initial bilirubin level was 11.71 mg/dL (2.03–31.5), which was distributed as follows: SPS (14.22 mg/dL), MPS (11.32 mg/dL), and SEMS (9.61 mg/dL) with *p* < 0.005. The percentage of patients who reached the target bilirubin level and the mean time in days to reach this target in the SPS, MPS, and SEMS groups were 35% (16.6 days), 85% (38.4 days), and 71% (19.52 days), respectively, with *p* < 0.005.

The success rate in achieving BT <2 mg/dL for SEMS, MPS, and SPS was 68.5% (24/35), 83.3% (5/6), and 41.1% (7/17), respectively. Although there was mainly divergence between SPS and MPS and SEMS, this difference was not significant (*p* < 0.093) (Table 2).

**Figure 1:**
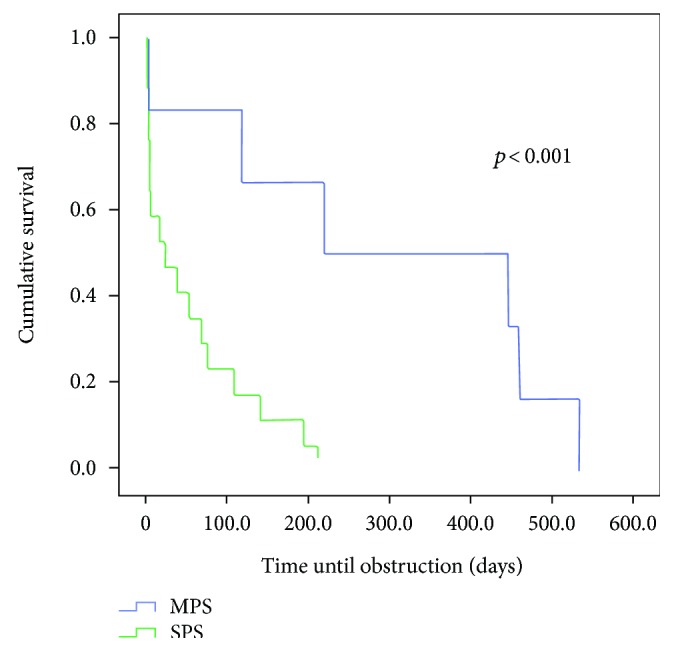
Comparison of the permeability time of patients undergoing SPS and MPS insertion (*p* < 0.001).

**Figure 2:**
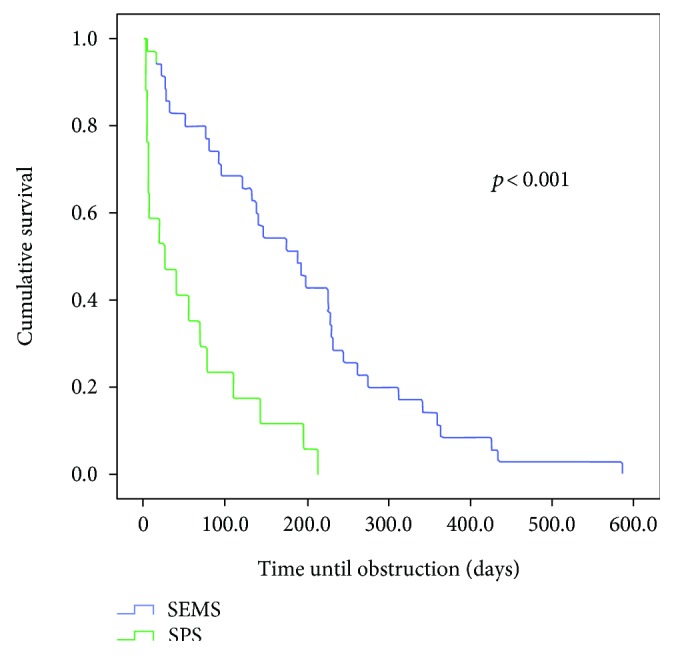
Comparison of the permeability time of patients undergoing SPS and SEMS insertion (*p* < 0.001).

**Figure 3:**
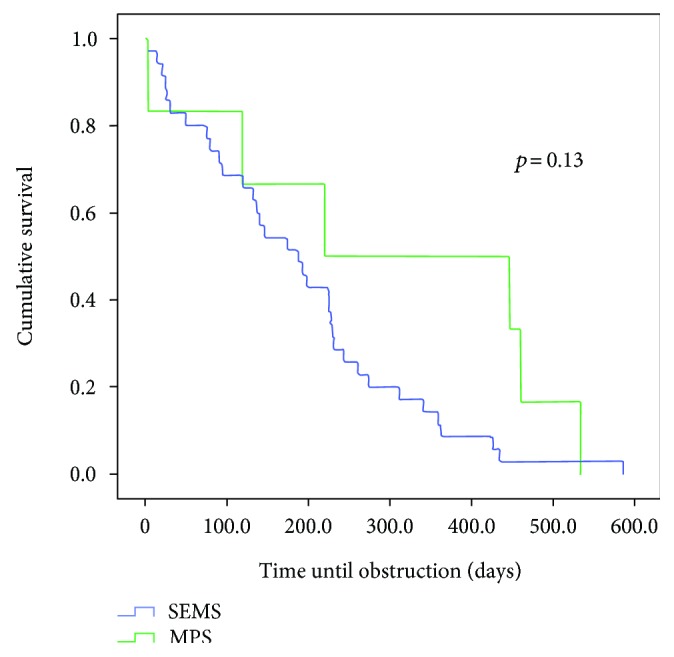
Comparison of the permeability time of patients undergoing MPS and SEMS insertion (*p* = 0.13).

**Figure 4:**
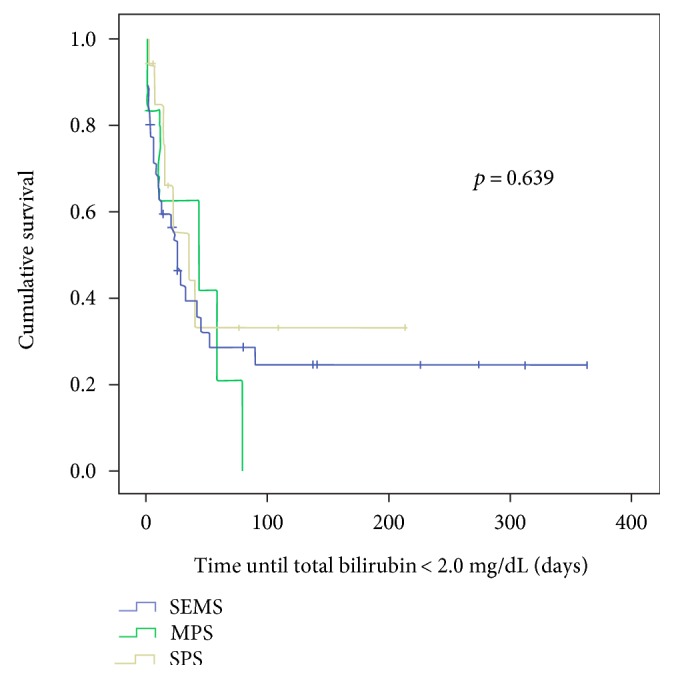
Comparison among the three groups in relation to the time in days to reach TB ≤ 2.0 mg/dL.

Comparing the survival curves with respect to obstruction, we observed a lower mean permeability of the SPS compared to the MPS with *p* < 0.003 and in relation to the SEMS group (*p* < 0.01) (Figures 1 and 2). However, when MPS versus SEMS was evaluated, no statistically significant difference was observed (*p* < 0.134) (Figure 3). There was no difference in the rate of fall of total bilirubin in those who reached TB <2 mg/dL regardless of the method used (*p* < 0.639) (Figure 4).

## 4. Discussion

The main cause of malignant biliary obstruction is pancreatic cancer. Of these patients, only 15 to 20% can be operated after staging obtained CT or MRI, and even after surgery, the 2-year survival varies around 10 to 20% [3, 4]. Pancreatic cancer is the fifth leading cause of death according to the CDC [5]. Those that can not be operated need palliative treatment to control complications inherent of the locoregional disease progression. Endoscopic biliary drainage is the principal method of treating jaundice in these patients [6, 7].

The main benefits of biliary drainage are relief of jaundice and allowance to chemotherapy in good clinical conditions. Most of the used regimens have hepatotoxic drugs, which can induce steatohepatitis or sinusoidal obstruction [8–10]. To be used to its full potential, it is necessary that the total bilirubinemia is up to 1.5 times above the normal value [11]. The choice of the TB limit <2.0 mg/dL as a mark of therapeutic success reflects the aggressive need to ensure good biliary drainage in relation to what the current chemotherapy regimens demand. However, the benefit of using bile duct stents is limited due to the occurrence of stent obstruction, patency time of the stent with worsening of jaundice, presence of cholangitis, and time (speed) for TB to reach adequate levels for initiation of chemotherapy on its full potential.

Our sample is comprised of patients with pancreatic adenocarcinoma (74.6%), metastasis (15.5%), and pancreatic neuroendocrine tumors (5.1%). Similar numbers have already been reported in the literature, corroborating the representativeness of our sample. The global percentage of patients who achieved the bilirubinemia target was 37.9%. This value differs from that previously found by Weston et al. of 76.2% when only patients with adequate follow-up were included in their series [2]. Observing only those with SEMS and MPS, the clinical success rate rises to 71% and 85%, respectively, and there is an apparent improvement in outcome (*p* = 0.09) (Table 2). Although the knowledge that such low levels of bilirubinemia are required for chemotherapy [11, 12], there is little reference in the literature to the quantity/percentage of patients achieving such a target. The only source that evaluates the time required to reach the proposed target is a study by Weston et al. [2] which evaluates the time it takes for 80% of patients to reach the target of 2 mg/dL. In this study, it is shown that patients with initial TB of less than 10 mg/dL usually take 2.7 weeks to reach the target and the same value for those with initial TB above 10 mg/dL is 5.6 weeks. The same work shows that the rate of fall of TB is independent of the chosen drainage method, which was corroborated by our casuistry.

The use of SEMS is more durable and requires fewer reinterventions when compared to the use of biliary drainage with SPS. While the use of SPS has an estimated mean permeability of 90 to 120 days, this estimate increases to 240–270 days for SEMS [13–16]. The main cause of obstruction in cases of SPS is the accumulation of debris in its interior, this being credited to its smaller diameter and the bacterial colonization that adheres to the internal wall of the stent when compared to SEMS. Hausegger et al., using SEMS, reported an occlusion rate of 33% with fully covered stent, but without a statistical difference between covered and uncovered [17]. The incidence of early obstruction is common among SPS plastic stents [1, 17, 18], which is a critical factor in choosing the drainage method in a patient who will receive chemotherapy with risks of hepatic failure or severe septic complications. In our study, the worst results regarding survival until stent obstruction were related to the use of SPS. We adopt an on demand regimen of sten exchange in patients with MDBO to avoid unnecessary procedures and hospital staying.

Several unsuccessful attempts were made to improve SPS drainage from higher caliber stents to stents with different formats [19, 20]. Tabibian et al. have shown that there is a bile flow between the stents when MPS are applied, even when some or all of them are obstructed [21]. In this same study, the author verified that the use of MPS allows a longer time of permeability, reducing the need for repeated endoscopic interventions in biliary stenoses after hepatic transplantation, but there is no evidence of this assertion in patients with malignant disease, which further corroborates the value of this presented study.

Lawrence et al. similarly obtained a longer permeability time by applying MPS (2 stents) in stenosis after surgery [22]. Despite this knowledge about MPS in benign stenoses, there is only one report of MPS in patients with MDBO, in which two prostheses were applied increasing the permeability time [23]. There are no data in the literature that compare the application of MPS to other endoscopic drainage methods.

The application of MPS in our evaluation proved effective. The combination of mean patency was similar to that of SEMS and the reduced cost of using MPS allows us to think of this as the best form of biliary drainage. The fact that there was no statistical difference in speed (time) to reach TB <2 mg/dL among groups is in line with previous research showing similarity in drainage quality among methods in the first month [24]. However, the use of TB <2 mg/dL as the object of evaluation reflects in a practical way the current need that a patient with MDBO has.

There are several limitations in the present study mainly due to its retrospective nature. The groups were uncontrolled, with a small sample in the SPS and MPS arms and the study was from a single center.

The rate of adverse events found, although similar to that reported in other studies, may be underestimated because of the retrospective nature of the study [1]. Another weakness of this study is the reduced number of patients in the SPS and MPS groups. The application of MPS in our institution is an exception being applied according to the preference of the endoscopist at the time of the procedure.

Based on the results of this study, the authors conclude that endoscopic biliary drainage is effective and presents a low rate of adverse events. The use of SPS was not effective in achieving the total bilirubin levels desired for chemotherapeutic treatment, and there was no statistical difference between the use of MPS, despite the small number of patients compared to the use of SEMS to reach satisfactory levels of bilirubin for the chemotherapeutic treatment, thus determining that the use of both SEMS and MPS can be performed in patients with MDBO.

## Figures and Tables

**Table 1:** Demographic findings of the patients and the etiological cause of MDBO.

Demographic findings	G1 (SPS)	G2 (MPS)	G3 (SEMS)	Total	*p*
Number of patients	17	6	35	58	—
Mean age	59.35 (15.51)	67.3 (4.59)	67.04 (10.28)		0.23
Gender (M/F)	8/9	2/4	10/15		0.99
Type of obstruction	—	—	—	—	—
Pancreatic cancer	11	4	29	44	0.52
Neuroendocrine tumor	2	0	1	3	0.46
Papillary carcinoma	0	1	1	2	0.99
Metastasis	—	—	—	—	—
Colon	2	1	2	5	0.46
Lung	0	0	1	1	0.99
Kidney	0	0	1	1	0.99
Rhabdomyosarcoma	1	0	0	1	0.36
Ovary	1	0	0	1	0.36

**Table 2:** Cross tabulation comparing therapeutic success between groups (*p* < 0.093).

TB ≤ 2 mg/dL	Yes (%)	No (%)	Total (%)
G1 (SPS)	7 (19.4)	10 (45.5)	17 (29.3)
G2 (MPS)	5 (13.9)	1 (4.5)	6 (10.3)
G3 (SEMS)	24 (66.7)	11 (50)	35 (60.3)
Total	36	22	58
